# 3D-printed composite scaffold with anti-infection and osteogenesis potential against infected bone defects

**DOI:** 10.1039/d2ra00214k

**Published:** 2022-04-08

**Authors:** Zewen Qiao, Wenping Zhang, Haifeng Jiang, Xiang Li, Weijun An, Haibo Yang

**Affiliations:** Department of Orthopedics, General Hospital of Ningxia Medical University Yinchuan 750021 China anweijun939@163.com qzw19820320@126.com +86-951-6743243; School of Mechanical Engineering, Shanghai Jiao Tong University Shanghai 200240 China

## Abstract

In the field of orthopedics, an infected bone defect is a refractory disease accompanied by bone infection and defects as well as aggravated circulation. There are currently no personalized scaffolds that can treat bone infections using local stable and sustained-release antibiotics while providing mechanical support and bone induction to promote bone repair in the process of absorption *in vivo*. In our previous study, rifampicin/moxifloxacin-poly lactic-*co*-glycolic acid (PLGA) microspheres were prepared and tested for sustained release and antibacterial activity. The composite scaffold of poly-l-lactic acid (PLLA)/Pearl had a positive effect on mechanics supports and promoted osteogenesis. Therefore, in this study, the personalized scaffolds of PLLA/Pearl were first prepared by 3D printing. Then, rifampicin/moxifloxacin-PLGA (RM-P) microspheres were loaded into the scaffold pores to prepare the PLLA/Pearl/RM-P scaffolds. In this *in vitro* study, we investigated the structural characteristics and cytocompatibility of 3D-printed composite scaffolds, which indicates the integrity of the components in the scaffolds. The PLLA/Pearl and PLLA/Pearl/RM-P composite scaffolds can promote adhesion, proliferation, and differentiation of human bone marrow mesenchymal stem cells. Moreover, a rabbit model of infected bone defects of the radius was established. PLLA, PLLA/Pearl, and PLLA/Pearl/RM-P scaffolds were implanted into the bone nidus. The therapeutic effect of the three scaffolds on the infected bone defects was evaluated through imaging and microbiological and histological analysis after surgery. Among the three scaffolds, only the PLLA/Pearl/RM-P scaffold had anti-infection and bone defect repair *in vivo*. 3D printing provides support for personalized scaffold structures, and composite materials ensure that the scaffolds exert anti-infection and bone repair effects. Our study suggests that the PLLA/Pearl/RM-P scaffold is a promising new material in the clinical treatment of infected bone defects.

## Abstract 2

Indication the mechanism of dual-functional scaffold in the treatment of infected bone defects.

## Graphical abstract

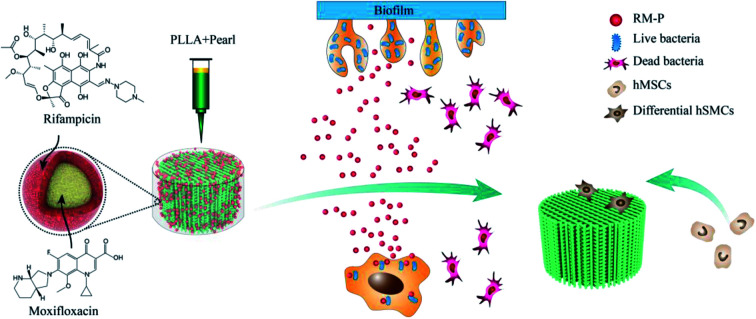

## 1 Introduction

In clinical practice, infected bone defects usually have a long disease course and poor prognosis, posing considerable challenges to doctors and patients.^1,2^ There are two main causes of infected bone defects. One is severe open fractures caused by high-energy injuries, and approximately 5–30% of such injuries will eventually develop into infected bone defects.^3^ The other is the increasing incidence of bone defects secondary to chronic bone infection caused by implant surgery in the later stage, which account for approximately 2–5%.^4^ Therefore, infected bone defects are still a common disease in orthopedic clinics. According to the principle of treating infection first and then repairing the defect, there are some clinical methods for treating infected bone defects such as open granule bone grafting, Masquelet technique, and bone transport surgery. However, these methods lack sustained and effective antibiotic therapy and are accompanied by repeated infection.^5–7^ It is difficult to recover from this illness. In the donor area, autologous bone is insufficient, and postoperative complications, such as pain and infection, are common. Patients are required to undergo multiple surgeries and incur huge costs, and the ossification and healing of the lesion area is a lengthy process.^2,8^ Therefore, developing a new treatment that can simultaneously heal the bone infection and defect, shorten the disease course, and improve disease prognosis is essential.

*Staphylococcus aureus* (SA) is a pathogen responsible for causing most cases of bone infection.^9^ It can form a biofilm on the endophytic and dead bone surfaces to protect itself from antibiotics and the immune system. Pathogenic bacteria in the bacterial biofilms can increase the minimum inhibitory concentration of antibiotics by 1000 times.^10,11^ Antibiotics are an essential component of bone infection treatment. However, owing to the pathological characteristics of significant ischemia at the site of bone infection and the existence of a blood–brain barrier, systemic antibiotic administration causes more toxic reactions in patients with other organs. The therapeutic effect is limited.^12^ In 1970, Buchholz and Engelbrecht combined bone cement with antibiotics in artificial joint replacement to prevent infection, which reduced the infection rate from 6% to 1.6%.^13^ To treat bone infection, antibiotic-loaded bone cement has two advantages. It can release a high concentration of antibiotics locally in the early stages and has a strong effect on bacteria in the biofilm. In addition, its hard plastic structure can occupy the cavity formed after the removal of the dead bone and further squeeze the living space of the bacteria. Furthermore, antibiotic bone cement has been reported used in the treatment of bone infection.^14,15^ Antibiotic bone cement is still commonly applied in clinical practice today. Many defects are associated with bone cement in terms of its use as an antibiotic carrier to treat bone infection because of its nature, such as the uncertainty of antibiotic release, limitation of antibiotic types, and nondegradable nature *in vivo*, which result in incomplete infection control, increase in drug-resistant strains and bacterial biofilm formation, and continuous adherence and proliferation of bacteria.^16–18^ Although there are many limitations, the local sustained release of antibiotics provides a new approach for the clinical treatment of bone infection.

At present, multimaterial composite scaffolds are an effective method for treating large bone defects.^10,19^ The properties of each material are critical in the preparation of an excellent scaffold. The resulting tissue-engineered bone must be biocompatible, contain bone-promoting factors, and have excellent mechanical properties.^20,21^ Polymers, such as polylactic acid and polyglycolic acid, are considered the most important for the preparation of tissue-engineered bone. They are easy to form and have good biocompatibility. However, they do not contain bone-promoting factors, and their mechanical properties are extremely different from those of bone tissue, thus limiting their use as bone scaffold materials alone.^22–24^ The nacreous layer is a bioceramic that is composed of calcium carbonate and conch hard protein, which has good biocompatibility. A recent study has shown that the organic matter in pearl powder is mainly composed of active components such as proteins and polysaccharide, which can improve the biocompatibility of scaffolds and promote osteogenesis.^25,26^ At present, it is a good composite material, which can be used to prepare tissue-engineered bone with polymer as the main component and a nacreous layer for bone induction and mechanical support. In the past decade, 3D printing technology has advanced considerably and is utilized in the field of medicine to a large extent.^27^ Its bioprinting technology provides an effective method for tissue engineering of fine internal structure and personalized external shape of bone.^28^

In this study, 3D printed porous poly-l-lactic acid (PLLA)/Pearl scaffolds were loaded with rifampicin/moxifloxacin-poly lactic-*co*-glycolic acid (PLGA) (R/M-P) microspheres to form PLLA/Pearl/RM-P scaffolds. The characterization and cytocompatibility of the scaffolds were investigated *in vitro*. The performance of scaffolds in treating infected bone defects was evaluated by microbiological, histological analysis, and imaging findings *in vivo*.

## 2 Material and methods

### 2.1 Materials

PLGA (LA/GA = 50/50, *M*_w_ = 9 × 10^4^) and PLLA (*M*_w_ = 17.9 × 10^4^) were purchased from Shandong Medical Instrument Co. Ltd.; Pearl powder was purchased from Hainan Finance Co. Ltd. (maximum particle size was 24.7 micron), moxifloxacin and rifampin were purchased from Sigma Company. Moxifloxacin was dissolved in deionized water (15 mg mL^−1^), and PLGA and rifampicin were dissolved in dichloromethane (DCM) at concentrations of 120 and 15 mg mL^−1^, respectively. The other reagents used in the experiment were purchased from Sigma and were not purified.

### 2.2 Preparation of RM-P microspheres, PLLA/Pearl scaffolds, and PLLA/Pearl/RM-P scaffolds

RM-P microspheres were produced using a double water–oil–water (W_1_/O/W_2_) emulsion by solvent emulsification–evaporation technique.^29^ Herein, the double water–oil–water (W_1_/O/W_2_) emulsion was composed of W_1_ (solution of moxifloxacin, rifampicin and PLGA in 900 μL acetonitrile), oil phase (petroleum ether and liquid paraffin) and W_2_ (aqueous solution of 1% PVA). W_1_ was added to 25 mL petroleum ether and liquid paraffin with stirring to form the organic phase (W_1_ : O). Then 40 mL 1% PVA solution was slowly added and stirred at 8000 rpm for 10 min to form a double water–oil–water (W_1_/O/W_2_). The emulsion phase was added to 800 mL 0.5% PVA solution and stirred at 6000 rpm for 3 h at room temperature. The RM-P microspheres were collected and freeze-dried at −80 °C for 24 h.

PLLA was accurately weighed and added in 1,4 dioxane and stirred with a magnetic mixer for 8 h until it slowly dissolved with concentration of 6% (m/v). Similarly, a uniform suspension of PLLA/Pearl with w/w (80/20) was prepared. The diameter of the scaffold was 5 mm, the height of each layer was 0.6 mm, and the total height was 10 layers. The prepared solution was added to the 3D-Bioplotter (EnvisionTEC GmbH, Germany) and heated to 150 °C at a pressure of 110 kPa and a nozzle speed of 18 mm s^−1^. After printing the scaffolds, they were placed in a freeze-drying machine for 24 h at −80 °C and for 2 days at room temperature to prepare PLLA and PLLA/Pearl scaffolds.^30^

Approximately 5 mg poloxamer was added into 10 mL of deionized water at 50 °C and stirred until it melted. In solution of poloxamer, 1 mg RM-P microspheres were uniformly dispersed to form RM-P microsphere suspension. The 3D-printed PLLA/Pearl scaffolds were placed in RM-P microsphere suspension and were vibrated in an ultrasonic instrument for 15 min. The scaffold was then freeze-dried at −80 °C for 24 h to obtain the PLLA/Pearl/RM-P scaffolds.

### 2.3 Scaffold characterization

The three types of scaffolds were dried with ethanol, and the surface of the scaffolds was sprayed with gold for 10 min. Scanning electron microscopy (SEM, S-3400N, Hitachi, Japan) was used to set the scaffolds' working voltage to 10 kV. The scaffolds' pore diameter, frame structure, and the presence of surface microspheres were observed.

The hydrophilicity of RM-P microspheres and pearl powder was measured by a water contact angle test (SPCA-X, HARKE, Beijing, China). The two materials were placed on the sample platform. A water contact instrument was used to detect the hydrophilicity of the two materials. Each material was measured three times in parallel to calculate the average water contact angle.

The thermogravimetric analysis (TGA) (TA Q-500, USA) was used to measure the specific gravity of RM-P and pearl in the scaffolds. The PLLA, Pearl power, and PLLA, PLLA/Pearl, PLLA/Pearl/RM-P scaffolds were placed in the TGA. The test conditions are as follows: the heating range is 30–800 °C, the heating rate is 10 °C min^−1^, and the nitrogen flow rate is 50 mL min^−1^.

### 2.4 Cell compatibility test

The scaffolds were sterilized in cobalt 60 using an 8 kGy irradiation intensity. The scaffolds were immersed in the culture medium for 6 h before the experiment to maintain humidity.^31^ The scaffolds were placed on a 24-well plate. Human mesenchymal stem cells (hMSCs) isolated before the experiment and cultured to the third generation were suspended in 5 mL α-MEM medium to form a single-cell suspension counted, and the cell concentration was adjusted to 3.0 × 10^4^ cm^−2^. The modified single-cell suspension was added to the surface of the scaffold. The cells were maintained as adhered to the surface of the scaffold as possible to facilitate the observation of cell morphology in the later stage. After the single-cell suspension was added, the scaffold was transferred to a constant temperature incubator for culture for 4 h. The α-MEM medium was added continuously to each well until the scaffold was completely covered. The solution was then transferred to a constant temperature incubator. The medium was completely drained after the first 24 h and was replaced with a new medium every 2 days.

The Cell Counting Kit (CCK)-8 was used to detect the proliferation activity of hMSCs. The scaffolds with adhered cells were transferred to the new 24-well plates after 3 and 7 days of culturing, respectively. The scaffolds were slowly rinsed with PBS solution three times, and the PBS solution was completely extracted before adding 500 μL medium and 50 μL CCK-8 reagent. Approximately 100 μL medium was poured out of each well into a 96-well plate, blown with a pipetting gun, and added to 3-wells of each type in parallel. The wavelength of the ELISA spectrophotometer was positioned at 450 nm for detection according to the standard of the CCK-8.

The cells were cultured on the scaffold for 3 and 7 days, respectively. Then, the scaffold was transferred to a new 24-well plate for cell lysis. The lysed cell fluid was then added to the centrifuge tube for centrifugation, then 100 μL of supernatant was added to the 96-well plate, and each sample was tested in triplicate. The alkaline phosphatase (ALP) activity was determined using the ALP microplate kit, and the ALP activity was determined using an enzyme-linked immunosorbent spectrophotometer with the wavelength of 520 nm adjusted according to the ALP kit standard.

The SEM was used to observe the surface cell morphology of scaffolds. Scaffolds cocultured with cells for 7 days were fixed with 2.5% glutaraldehyde and 2% paraformaldehyde for 3 h, respectively, and then dehydrated with a series of concentration gradient alcohol (30%, 50%, 70%, 90%, and 100%). After freeze-drying, the surface of the scaffold was sprayed with gold, and the cell morphology of the scaffold was observed using SEM.

### 2.5 Establishment and scaffold implantation of infected bone defect model in rabbits

To evaluate the anti-infection and osteogenic effects of PLLA/Pearl/RM-P scaffolds on rabbits with infected radius bone defects. The experiments performed in rabbits were reviewed and approved by the Ethics Committee of Ningxia Medical University General Hospital (2020-305), and all the operations were performed in accordance with the Ethics Committee. To establish a model of infected bone defect, 27 bone mature New Zealand white rabbits (3.0–4.0 kg) were selected. Before the experiment, pentobarbital sodium was diluted with 0.9% NaCl to 1% of the original solution. The radius of the rabbit was selected as the modeling site. The rabbits were anesthetized intraperitoneally at a dose of 100 mg kg^−1^. After administering anesthesia, the skin was prepared on both forelimbs of rabbits, and the radius was accurately positioned. The middle length of the radius was exposed up to approximately 2 cm. The truncated length of the radius was measured and marked as 6 mm. The radius was truncated at the marked site with a miniature orthopedic pendulum saw to avoid damage to the ulna and peripheral vascular nerves. Simultaneously, normal saline was injected into the pendulum saw to avoid thermal damage to the bone. The osteotomy area was rinsed with normal saline repeatedly. After opening the medullary cavity at the distal and proximal ends of osteotomy with a 1.0 Kirschner wire, the previously prepared bacterial fluid (ATCC25923,1 × 10^6^ CFU mL^−1^) was injected into the distal and proximal medullary cavities, and the simple PLLA, PLLA/Pearl, and PLLA/Pearl/RM-P scaffolds were implanted into the defect area. Layers of periosteum, muscle, and skin were sutured. After surgery, the rabbits were placed in the lateral decubitus position in the cage, and the animals were irradiated with a baking lamp to avoid hypothermia while anesthetized, and rabbits were observed to prevent asphyxia while anesthetized until the animals fully recovered. Postoperative forelimbs were allowed to move freely. Postoperative rabbits could move, drink and eat freely in the cage. Routine antibiotic treatment was not given before and after surgery.

#### 2.5.1 Postoperative imaging evaluation

Three rabbits from each group were randomly selected for imaging evaluation. At the 4th and 8th week after surgery, X-rays of the surgical site of the selected rabbits were examined to evaluate the severity of osteomyelitis in each group. Pentobarbital sodium diluent (1%) was injected intraperitoneally to keep the rabbits in a resting state during the examination. The results were reported in a double-blinded condition by three experienced radiologists. Imaging judgment criteria were based on the standard of X-ray parity for bone infection proposed by Lucke:^32^ the degree of soft tissue swelling in the experimental area, the periosteal reaction, bone destruction, and the presence of deformity. A micro-computed tomography (Micro-CT) of the surgical area was performed 8 weeks after the operation, and the obtained images were reconstructed by software. The images were analyzed using a cross-section of the shaft's long shaft. The scan data were used to calculated the bone volume/total volume (BV/TV) × 100%.

#### 2.5.2 Evaluation of viable bacteria

At the 4th week after surgery, three rabbits from each group were randomly selected to evaluate the viability of bacteria at the surgical site as previously described.^33^ The implanted scaffold was removed under aseptic conditions for the plate rolling experiment, and the culture plate was placed in a 36 °C incubator for 24 h. Simultaneously, approximately 5 mm of the bones at both ends of the scaffold were taken, placed in sterile grinding dishes filled with liquid nitrogen, and samples were ground into powder under aseptic conditions. Then, the bone powder was placed in a sterile test tube, and PBS solution was added to the vortex shock, fully mixed, and diluted 100 times. On the bacterial culture plate, 100 μL were smeared and incubated for 24 h at 36 °C.

#### 2.5.3 Histological analysis

At the 8th week after surgery, three rabbits from each group were sacrificed through the ear vein air embolization. With the original surgical incision for the center, the upper and lower incisions were extended by approximately 3 cm each, and the incision was cut layer by layer. The micropendulum saw was used to cut the bone at approximately 2 cm above and below the scaffold, and the attached muscles and periosteum of the diaphysis were scraped off, and the site of interest was removed. For 48 h, each group's labeled specimens were immersed in a 10% formalin fixation solution. Remove the specimen and rinse the residual fixed solution with running water. For the dehydration treatment, the specimens were placed in ethanol concentrations of 70%, 80%, and 90%, respectively. The soaking time of each concentration was approximately 2 h. After that, it was immersed in a solution (a mixture of methyl methacrylate, dibutyl phthalate, and xylene) for 2 weeks. It was then soaked in methyl methacrylate (PMMA) solution for 48 h and decalcified in a rapid decalcification agent for 2 weeks. For curing, the container containing the specimen and embedding solution was placed into a 37 °C constant temperature water bath. After curing, Masson's trichrome and H&E staining were used to assess bone infection and new bone formation.

### 2.6 Statistical analysis

For statistical analysis, SPSS19.0 software was used. The mean ± standard deviation is used to express all quantitative data. *In vitro* experiments were repeated three times, and three samples were collected from each group *in vivo* experiments. One-way ANOVA *t*-test and Mann–Whitney *U* test were used to compare the groups. A *P* value of <0.05 indicated a significant difference among all groups, as shown in the chart.

## 3 Results and discussion

### 3.1 Morphological characteristics

**Fig. 1:**
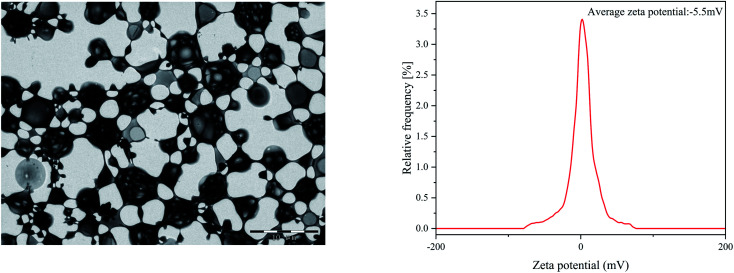
The TEM image of RM-P microspheres (A) and its zeta potential (B).

**Fig. 2:**
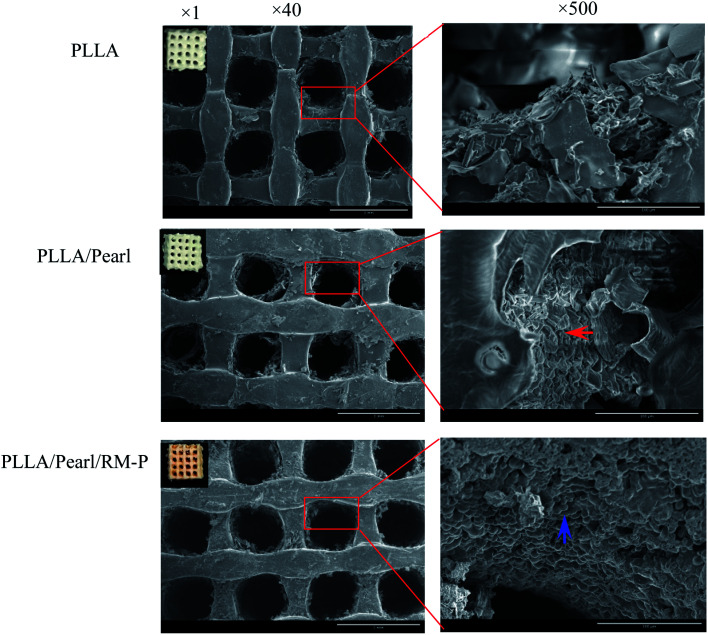
There are the morphological characterization and SEM images of composite scaffolds. The red arrow marks the pearl powder on the surface of the scaffold, and the blue arrows indicate intact RM-P microspheres on the surface of the scaffold.

Present studies are mainly focused on the development of multimaterial composite multifunctional bone scaffolds based on clinical characteristics of the disease. In this study, PLLA, antibacterial microspheres, and pearl powder were combined with 3D printing technology and freeze-drying method to construct a dual-function bone scaffold, which had anti-infection and osteogenic potential, for use in clinical practice to treat infected bone defects. The RM-P microspheres showed an average hydrodynamic size of 16.62 μm.^29^ The morphology of RM-P microspheres were examined by transmission electron microscopy (TEM) and its zeta potential was −5.5 mV (Fig. 1). The general morphology and characteristics of the scaffold under SEM are shown in Fig. 2. The biological 3D-printed PLLA scaffold is a multilevel scaffold formed by connecting multiple square pores having a uniform and regular frame. The frame structure of the scaffold and the material properties of PLLA and pearl powder provide excellent mechanical properties on the scaffold, as well as space for loading the RM-P microspheres later. In this study, RM-P microspheres were freeze-dried and added to the scaffold. Overall, the color of the PLLA/Pearl scaffold showed no remarkable change, and the color of PLLA/Pearl/RM-P was uniformly similar to that of RM-P microspheres, indicating that RM-P microspheres were uniformly distributed in the scaffold. Under SEM, pearl powder was found on the surface of the scaffold, RM-P was filled with the PLLA/Pearl/RM-P scaffolds, and the RM-P microspheres were spherical and intact. It is essential for the RM-P microspheres to remain intact in the scaffold so as to ensure the stable and sustained release of two antibiotics for the effective treatment of bone infections.

**Fig. 3:**
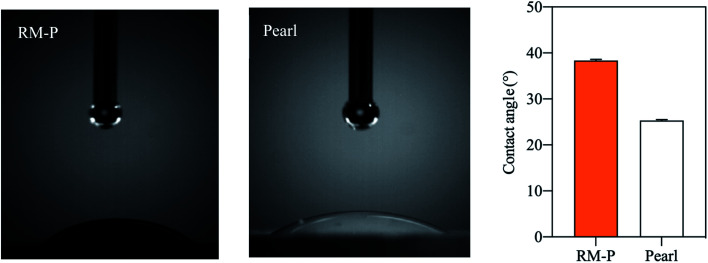
Water contact angle on RM-P and Pearl power.

The hydrophilicity of each material in the composite scaffold is used to determine the hydrophilicity of the scaffold.^34^ The water contact angle of the RM-P microspheres and the pearl powder was used to evaluate the hydrophilicity of the PLLA/Pearl/RM-P scaffold. The contact angle of RM-P microspheres was 38.4° ± 0.3°, and the contact angle of pearl powder was 25.3° ± 0.2° (Fig. 3). The water contact angle of both materials was less than 90°, indicating that they have good hydrophilicity. The smaller the water contact angle, the higher is the hydrophilicity, which is beneficial for cell adhesion and proliferation. The larger the water contact angle, the higher is the hydrophobicity and thus less conducive to cell adhesion and proliferation.^10,35^ The results of the measurements showed that pearl powder had higher hydrophilicity than RM-P microspheres, and there was a statistical difference between them. The results showed that the PLLA/Pearl/RM-P scaffolds with pearl powder had high hydrophilicity.

**Fig. 4:**
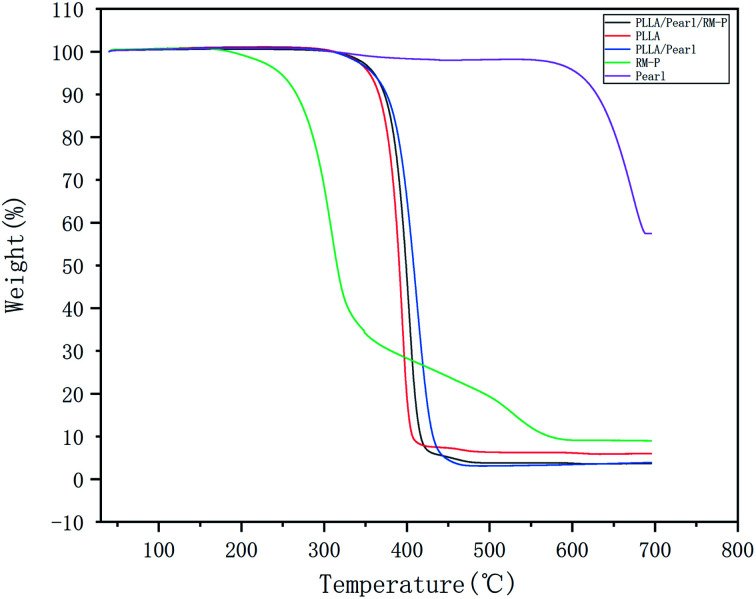
The TGA curve shows the content of various materials in the composite scaffold.

The TGA results are shown in Fig. 4. These five materials do not undergo weight loss below 200 °C, indicating that the thermal stability of each material is good. Pure RM-P microspheres underwent a weight loss of nearly 90% between 200 °C and 680 °C, whereas that undergone by pearl powder was nearly nonexistent below 680 °C. Among the three scaffolds, the weight loss in PLLA/Pearl/RM-P appeared first, which corresponded to the fact that the heat resistance of RM-P microspheres was lower than that of PLLA and pearl powder. At 450 °C, the weight loss in PLLA/Pearl/RM-P was 75%, which was mainly caused by the thermal decomposition of PLLA and RM-P microspheres. Because pearl powder had a higher heat resistance than the other two scaffolds, the weight loss temperature of PLLA/Pearl was slightly higher, and the actual weight loss was 65% at 450 °C.

### 3.2 Cell compatibility

**Fig. 5:**
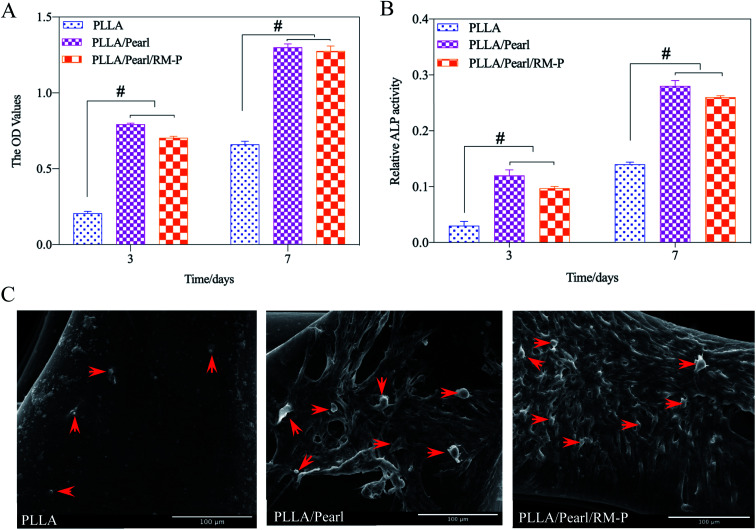
(A) and (B) show the OD and ALP values measured by CCK-8 kit and ALP kit after the scaffolds were co-cultured with hMSCs for 3 days and 7 days. (C) how the appearance of adherent cells (red arrow) on the surface of PLLA, PLLA/Pearl and PLLA/Pearl/RM-P under SEM, respectively. Scale bar = 100 μm (C). Date are represented as means ± SD (*n* = 3). Significant differences are indicated as #*P* < 0.05.

To evaluate the cytocompatibility of the scaffolds, hMSCs were cocultured with these three scaffolds. A CCK-8 was used to determine the adhesion and proliferation of hMSCs cultured on three scaffolds for 3 and 7 days. The OD values at each time point are presented in Fig. 5A. Although the three scaffolds showed hMSC adhesion, the two scaffolds containing pearl powder showed increased hMSC adhesion, and as the coculture time increased, the proliferation of hMSCs on PLLA/Pearl and PLLA/Pearl/RM-P scaffolds showed more proliferation than the PLLA scaffold (*p* < 0.05). All three scaffolds had good cytocompatibility. The active compounds in pearl powder were found to promote hMSC adhesion and proliferation, which is consistent with other studies.^36,37^

To evaluate cell differentiation on the scaffold surface, the ALP activity of hMSCs cultured on the scaffold surface for 3 and 7 days was determined (Fig. 5B). On the 3rd and 7th days, no statistical difference was noted between the two. After 7 days, the ALP activity of the two scaffolds with pearl powder was significantly higher than that after 3 days. At the same time point, the ALP activity of PLLA/Pearl and PLLA/Pearl/RM-P was also significantly higher than that of PLLA. Thus, the PLLA scaffold exhibited good cytocompatibility and could promote cell adhesion. However, pearl powder showed better differentiation of hMSCs than PLLA.

SEM was used to observe the morphology of adherent cells on the surface of scaffolds (Fig. 5C). Fewer adherent cells were noted on the surface of the PLLA scaffold, whereas the number of adherent cells on the surface of the other two types of scaffolds containing pearl powder were significantly higher than those observed on the surface of the PLLA scaffold. Some cells were found to have protruded pseudopodia. Thus, pearl powder was found to promote cell adhesion and differentiation, whereas RM-P microspheres did not affect cell adhesion and differentiation.

### 3.3 Evaluation of treatment on infected bone defects *in vivo*

#### 3.3.1 Imaging evaluation

**Fig. 6:**
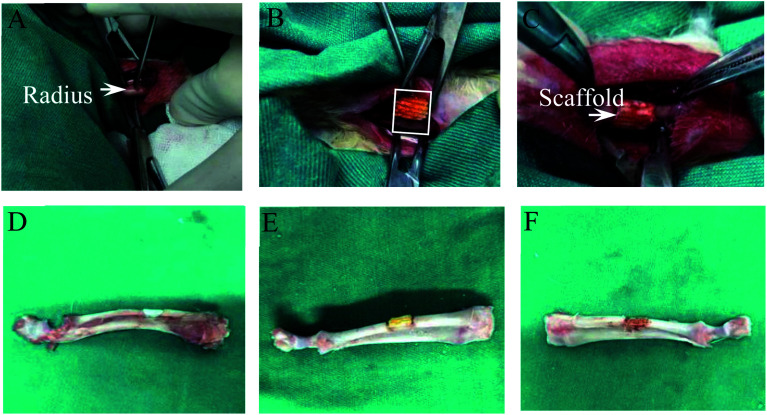
(A) is the exposed middle segment of rabbit radius, (B) is the length of osteotomy determined according to the length of scaffold, (C) is the implantation of scaffold after osteotomy. (D)–(F) are the appearance of implanted PLLA, PLLA/Pearl and PLLA/Pearl/RM-P scaffolds.

As shown in Fig. 6, the defect was located in the middle radius with a length of 6 mm. The scaffold was tightly embedded in the defect area. X-ray examination showed an absence of development in the scaffold. Because the ulna is the main bone of the upper limb of the rabbit, choosing the radius as the modeling area neither affects the normal activities of the rabbit nor necessitates the requirement of other metal implants to reduce artifact interference in imaging examination. The rabbits cannot repair a bone defect that is more than 1.5 times the diameter of the backbone.^38^ The diameter of the rabbit radius is generally 3 mm, so the length of radius defects in the experiment was set as 6 mm.

**Fig. 7:**
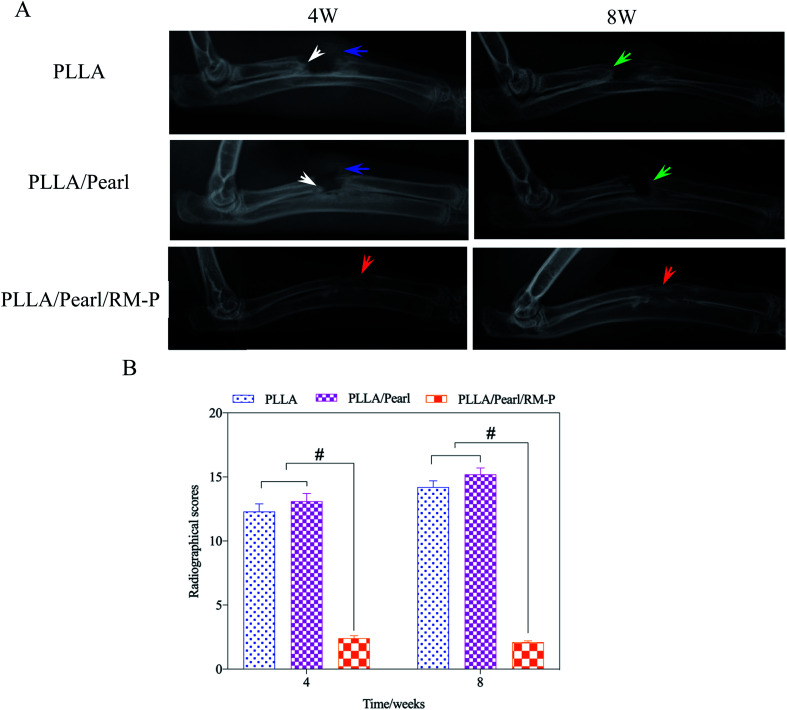
(A) X-rays of rabbit's upper limber with implantation of composite scaffold obtained at week 4 and 8 post-operatively. There are soft tissue swelling (blue arrow) and dead bone formation (white arrow) in the PLLA and PLLA/Pearl group. However, there are no signs of infection in the PLLA/Pearl/RM-P group, and some bone healing was observed (red arrow). Partial reactive callus and bone resorption appeared at week 8 post-operatively (green arrow). Bone union was achieved in PLLA/Pearl/RM-P group. (B) shows Lucke's scores at 4th and 8th week for the three groups. Date are represented as means ± SD (*n* = 3). Significant differences are indicated as #*P* < 0.05.

The clinical imaging manifestations of bone infection are gradually aggravated from surrounding soft tissue swelling and abscess formation to periosteum reaction, bone destruction, and dead bone formation.^39,40^ The results are shown in Fig. 7A. At the set time points (4 and 8 weeks) after surgery, an X-ray of three groups of New Zealand white rabbits implanted with different scaffolds was examined. After 4 weeks, soft tissue swelling and mass shadow were observed around the scaffolds in the PLLA and PLLA/Pearl groups, and periosteal reaction occurred around the radial of rabbits. At the contact between the scaffold and diaphysis, irregular bone resorption and patchy sclerotic dead bone were observed, which showed obvious signs of chronic osteomyelitis.^41^ However, there were no signs of osteomyelitis, such as periosteal reaction, bone absorption, and dead bone formation, on X-ray after 4 weeks of PLLA/Pearl/RM-P scaffolds implantation. New bone formation was observed in the junction area, and bone ingrowth was observed in the scaffold, indicating that no bone infection developed in the PLLA/Pearl/RM-P group in the 4th week. The infection signs in the PLLA group and PLLA/Pearl groups were worse in the 8th week than in the 4th week. The swelling and shadow of the surrounding soft tissues were reduced caused by the pus discharge from the infected sinus. The periosteal reaction was aggravated, and the formation of dead bone increased. The range of new bone formation in the PLLA/Pearl group was increased compared with that in the 4 weeks group, but it was still irregular, and there was no sign of healing with the scaffolds, and no bone connection was formed in the defect area. After 8 weeks, the PLLA/Pearl/RM-P group showed no signs of osteomyelitis, such as periosteal reaction, bone resorption, or bone malformation, and the formation of new bone was considerably increased. The bone was fused with the scaffold, and the density inside the scaffold increased, indicating that the amount of bone growth was increased and the defect area was completely repaired.

Simultaneously, X-ray manifestations of each group were scored according to Luke's osteomyelitis imaging evaluation system to quantify the degree of bone infection (Fig. 7B). In the 4th week, the imaging scores of the PLLA and PLLA/Pearl group were significantly higher than those of the PLLA/Pearl/RM-P group. After 8 weeks, the imaging scores of the PLLA and PLLA/Pearl groups were increased further, indicating further aggravation of infection. PLLA/Pearl/RM-P group showed no significant changes or imaging manifestations of infection.

**Fig. 8:**
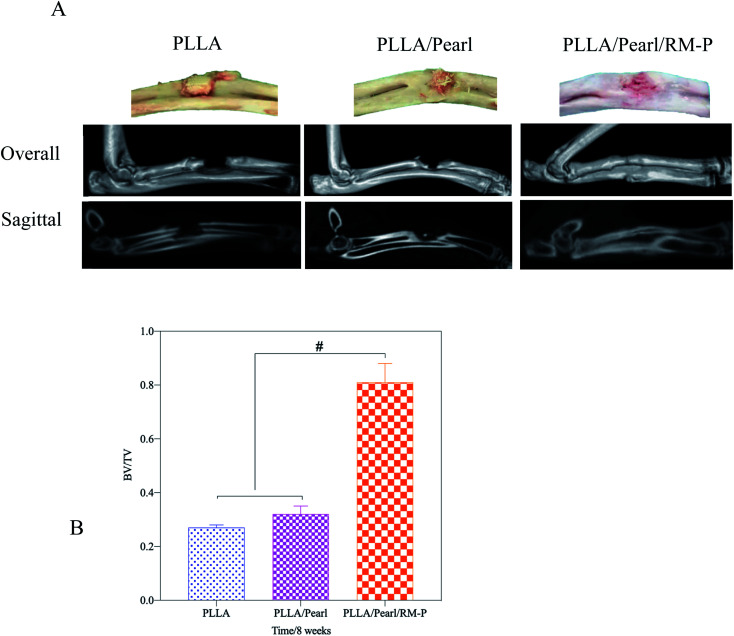
(A) Specimens and 3D micro-CT images of the rabbit's upper limb obtained at week 8 post-operatively. (B) Bone volume/total volume (BV/TV) of interested region evaluated by micro-CT. Date are represented as means ± SD (*n* = 3). Significant differences are indicated as #*P* < 0.05.

Micro-CT was used in the 8th week to evaluate the healing of the bone defect (Fig. 8). In the PLLA and PLLA/Pearl groups, osteomyelitis was observed at the defect site. Irregular bone formation was observed in both groups, but there was no union at the defect site. PLLA/Pearl/RM-P group showed no signs of infection, and new bone grew into the scaffold, forming a continuous healing bone at the bone defect site. Further morphological measurements were made on the lesion area to determine the new bone formation in the three groups of scaffold implantation areas. The BV/TV of the PLLA scaffold group was lower than that of the PLLA/Pearl group, and most of them showed reactive new bone formation caused by infection, with no statistical difference (*P* > 0.05). The BV/TV of the PLLA/Pearl/RM-P group was significantly higher than that of PLLA and PLLA/pearl powder groups, and more bone trabeculae were formed in the scaffold (*P* < 0.05). In chronic infections, new bone may form as infected callus, which is mainly composed of fragments of the damaged periosteum and irregular in shape, thus hindering the formation of a continuous callus.^42^ In this study, a callus formation was also observed in the two groups without RM-P microspheres, but the infection callus was dominant, and bone repair was not formed. When PLLA/Pearl/RM-P scaffolds were released to control infection, new bone was formed, and bone grew into the scaffold to achieve bone healing. Infection control is critical in the repair of infected bone defects.

#### 3.3.2 Evaluation of viable bacteria

**Fig. 9:**
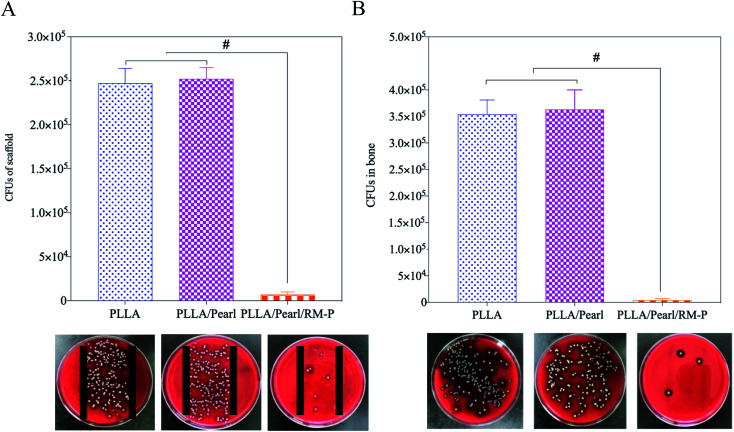
(A) The number of viable bacteria on the scaffold surface, and (B) the number of viable bacteria in adjacent bone of scaffold at week 8 post-operatively. Date are represented as means ± SD (*n* = 3). Significant differences are indicated as #*P* < 0.05.

In this study, viable bacteria on the surface of the scaffold implanted in animals were quantitatively detected and counted using the scaffold rolling plate experiment. The scaffolds were removed and implanted under aseptic conditions in each group 8 weeks later. After rolling the three scaffolds, the culture plates were placed in a 36 °C incubator for 24 h (Fig. 9A). The number of viable bacteria was higher in the PLLA and PLLA/Pearl groups without RM-P microspheres than in the PLLA/Pearl/RM-P group, indicating a significant statistical difference. The results showed that the bacteria in the first two groups survived adequately, and the scaffolds had no effect on the bacteria. The PLLA/Pearl/RM-P group killed the bacteria on the surface of the scaffold caused by the gradual release of antibiotics in the antibacterial microspheres, and nearly no bacteria survived after 8 weeks. The bacteria contained in the radius of approximately 0.5 mm on both sides of the scaffold were also quantified in the study. As shown in Fig. 8b, the number of bacteria per gram of bone tissue in the group without RM-P microsphere scaffolds was significantly higher than that in the group with RM-P microsphere scaffolds (Fig. 9B). The scaffolds without RM-P microspheres did not exert any antibacterial effect, and the bacteria had already entered the bone tissue.

The bacteria adhered to the internal plant surface or the ischemic site after SA was inoculated into the scaffold and the radial marrow cavity, which was the initial stage of infection.^43^ The number of bacteria exceeded the immune capacity of the body and gradually survived to form a bacterial biofilm. After 4–6 h of bacterial adhesion to the internal plant surface, biofilms can form and gradually develop to cause bone infection.^44^ SA in the biofilm can escape immunity, reproduce, and give rise to a local inflammatory reaction. In the absence of effective antibiotic intervention, late abscess formation and bone destruction will occur. This study showed that the PLLA/Pearl/RM-P scaffold killed the inoculated bacteria because of the slow release of antibiotics, and no bacterial growth was observed in the bone or on the surface of the scaffold. In the other two groups, bacteria proliferated on the surface of the scaffold and in the bone marrow cavity, resulting in bone infections.

#### 3.3.3 Histological evaluation

**Fig. 10:**
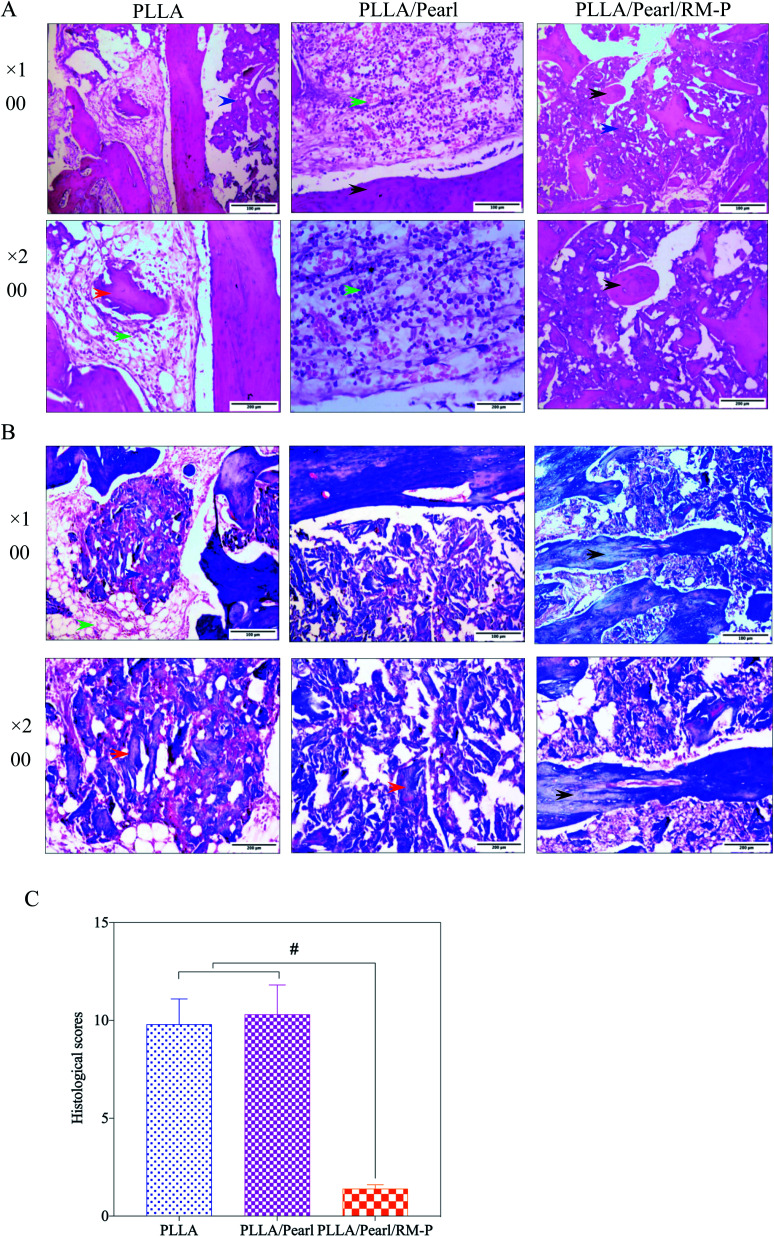
(A) H&E and (B) Mason staining was performed at the interface between the scaffold and bone 8 weeks after surgery. Pus cells (green arrow), dead bone (red arrow), and scaffold tissue without bone in growth (blue arrow) were observed in the PLLA and PLLA/Pearl groups. In the PLLA/Pearl/RM-P group, new bone in growth was seen in the scaffold (black arrow). (C) The histological scores of PLLA/Pearl/RM-P group was significantly different from that of the other two groups. Date are represented as means ± SD (*n* = 3). Significant differences are indicated as #*P* < 0.05.

Pathological examination of focal tissue is an important method for the diagnosis of bone infection.^45^ Infected bone tissue causes osteoclast activation, formation of pus cell aggregation in the focal area and bone absorption, and other pathological changes, which are pathological signs of bone infection.^46^ In this study, hematoxylin–eosin (H&E) and Mason staining were performed on the adjacent bone junction area and scaffold sections of the three groups at 8 weeks to observe the pathological changes. In the PLLA and PLLA/Pearl groups, many pus cells were found to be immersed in the adjacent bone interface area and the scaffold using the two staining methods (Fig. 10A). A portion of the dead bone could be observed in the interface area. Bone destruction and absorption, as well as a small amount of reactive new bone near the bone edge, were observed, suggesting that bacteria proliferate and cause chronic osteomyelitis. In the PLLA/Pearl/RM-P group, no pus cell aggregation was noted, and a lot of new bone was formed and passed through the junction area. The amount of bone that grew at the edge of the scaffold was higher than that noted in the center of the scaffold, and fusion was observed in the center.

The histological scores of the three groups of pathological images were also quantified using Lucke's bone infection histopathological evaluation system (Fig. 10B).^33^ The PLLA/Pearl/RM-P group showed no signs of infection, and Lucke's score was significantly lower than that noted for the other two groups (*p* < 0.05). There was no difference in scores between PLLA and PLLA/pearl powder groups (*p* > 0.05). The results showed that the PLLA/Pearl/RM-P group had a better anti-infection ability and certain osteogenic ability than the other two groups.

## 4 Conclusion

**Fig. 11:**
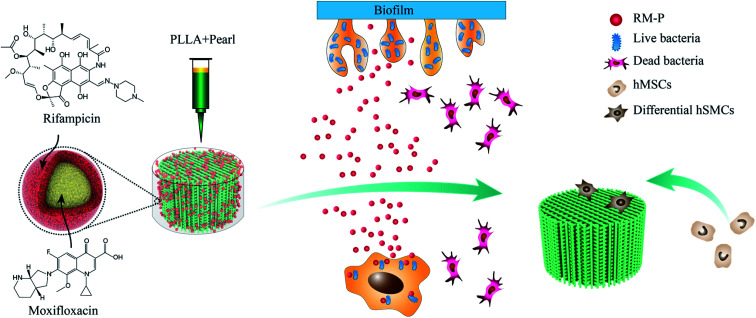
Indication the mechanism of dual-functional scaffold in the treatment of infected bone defects.

In this study, as shown in Fig. 11, RM-P microspheres loaded with rifampicin and moxifloxacin antibiotics were freeze-dried into a 3D-printed PLLA/Pearl scaffold. A dual-function bone scaffold having anti-infection and osteogenic potential was prepared for the clinical treatment of infected bone defects. Two antibiotics can be released smoothly through microspheres in the early stages of scaffold implantation to kill bacteria and treat bone infections. In the later stage, bone defects can be repaired using the scaffold's guiding effect and the pearl powder's osteogenesis promoting effect. *In vitro*, PLLA/Pearl/RM-P scaffolds demonstrated good antibacterial activity while also promoting bone cell adhesion and proliferation. The scaffold demonstrated excellent anti-infection and bone repair properties. Finally, this novel infection-resistant bone scaffold has significant potential for treating infected bone defects, and it may function as a new material and treatment approach for the clinical treatment of such diseases.

## Conflicts of interest

The authors declare that there is no conflict study.

## Supplementary Material
